# Perioperative carbohydrate loading improves comfort without adverse metabolic or neonatal effects in gestational diabetes mellitus

**DOI:** 10.1016/j.isci.2026.117488

**Published:** 2026-09-10

**Authors:** Siqi Liu, Yuxiang Bai, Xiaochang Yang, Longqiong Wang, Hongli Li, Guangmin Song, Ruohan Hu, Tongzhi Wu, Xiaoqing Tan, Qingrong Wu, Hua Zhang, Chang Chen

**Affiliations:** 1 Department of Obstetrics and Gynecology, The First Affiliated Hospital of Chongqing Medical University, Chongqing, China; 2 Chongqing Key Laboratory of Maternal and Fetal Medicine, Chongqing Medical University, Chongqing, China; 3 Medical School, University of Otago, Dunedin, New Zealand; 4 Centre for Research Excellence in Translating Nutritional Sciences to Good Health, School of Medicine, Adelaide University, Adelaide, SA, Australia; 5 Chongqing General Hospital, Chongqing University, Chongqing, China; 6 Fuling Hospital Affiliated to Chongqing University, Chongqing, China; 7 College of Pharmacy, Chongqing Medical University, Chongqing, China

**Keywords:** cesarean section, gestational diabetes mellitus, preoperative loading, enhanced recovery after surgery, metabolomics

## Abstract

Preoperative carbohydrate loading has been known to overcome discomfort and complications associated with cesarean section, but there is a scarcity of evidence on its safety and benefits in patients with gestational diabetes mellitus (GDM). In this study, pregnant women with normoglycemia and GDM were recruited and grouped according to their nutritional choice prior to cesarean delivery. Blood samples and clinical data were collected, and maternal comfort scores based on self-reports and neonatal data recorded. Metabolomic profiling was performed to explore underlying metabolic responses. Results showed perioperative carbohydrate loading significantly reduced thirst, hunger, and anxiety compared with fasting controls in both the normal and GDM groups. The two groups showed no significant differences at any time point in terms of glycemic measures, inflammatory markers, or maternal and neonatal outcomes. Metabolomic analysis revealed distinct but partially overlapping alterations in metabolic pathways at 1 h before surgery between the two groups.

## Highlights

• Carbohydrate drink before cesarean section improved comfort in women with GDM
• It did not adversely affect maternal or neonatal outcomes
• It affects fatty acid biosynthesis in both GDM and normoglycemic pregnancies
• It altered bile acid metabolism in normoglycemic but not GDM pregnancies

## Graphical abstract

**Figure:**
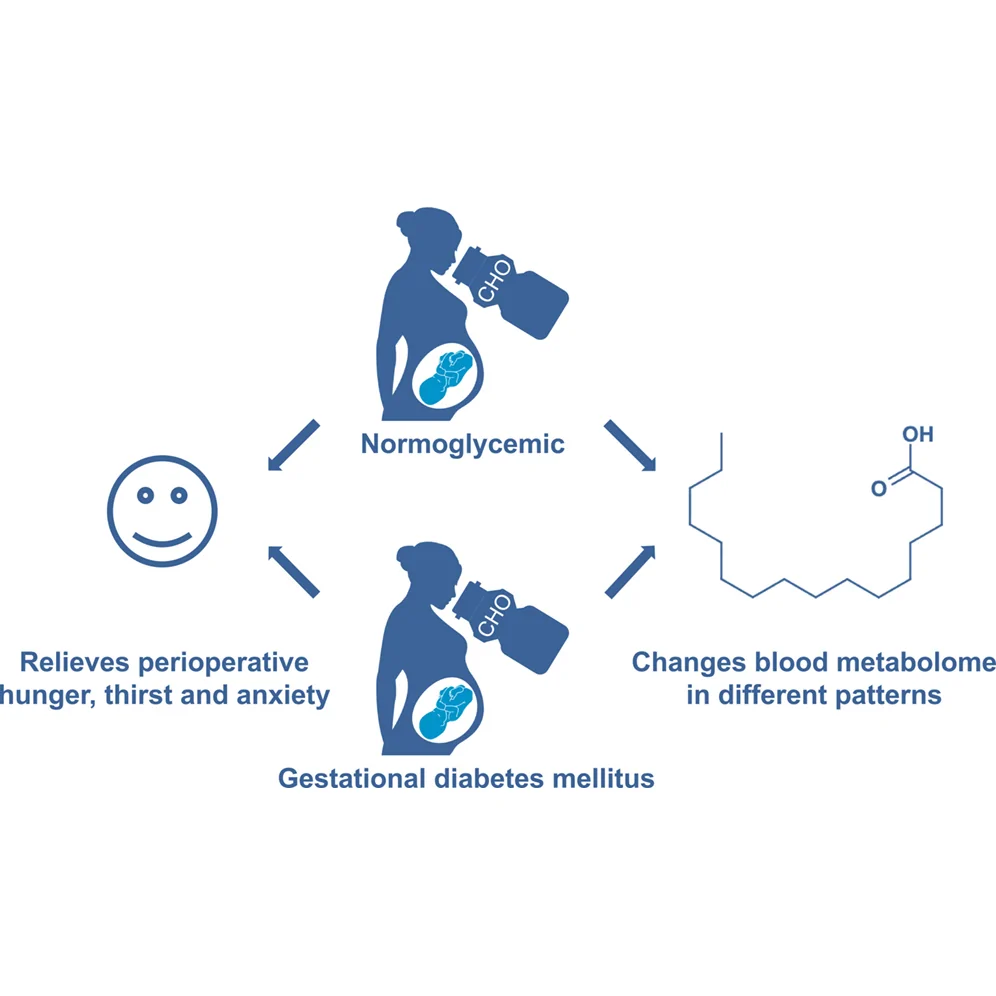

## Teaser

Endocrinology; Health sciences; Internal medicine; Medical specialty; Medicine; Reproductive medicine

## Introduction

Cesarean section serves as a primary intervention for managing critical obstetric emergencies, enabling timely delivery in life-threatening maternal and fetal situations. It has become the most frequently performed major surgical procedure worldwide.^1^ Recent epidemiological data indicate that 21.1% of all births globally are now delivered by cesarean section, a figure projected to rise to 28.5% by 2030.^2^

Traditional preoperative management for cesarean section typically mandates fasting from midnight before the procedure to reduce intraoperative aspiration risk. However, growing evidence associates prolonged fasting with several adverse effects, including elevated maternal urinary ketone levels,^3^ increased incidence of postoperative abdominal distension and vomiting,^4^ impaired postoperative recovery,^5^ and a higher risk of neonatal hypoglycemia.^6^

Enhanced recovery after surgery (ERAS) is an evidence-based, multimodal perioperative care approach designed to minimize surgical stress and accelerate functional recovery.^7^^,^^8^ It has been increasingly adopted across various clinical specialties in recent years.^9^^,^^10^^,^^11^^,^^12^ A central element of the ERAS protocol involves optimized preoperative fasting strategies, particularly shortening the preoperative fasting period and implementing preoperative carbohydrate loading—ingestion of clear carbohydrate-rich fluids up to 2 h before elective surgery.^13^ These measures have been shown to confer significant clinical benefits, including reduced postoperative nausea and vomiting, attenuated insulin resistance, and decreased inflammatory stress responses, ultimately leading to improved patient outcomes.^14^

Given these advantages, ERAS principles are gaining traction in the preoperative management of cesarean section. Emerging studies suggest that preoperative carbohydrate intake before cesarean delivery improves thermoregulatory stability during anesthesia,^15^ alleviates preoperative discomfort (thirst, hunger, and anxiety), mitigates postoperative insulin resistance,^16^ shortens the time to colostrum production, and reduces postpartum hemorrhage,^17^ collectively enhancing the perioperative experience.

Gestational diabetes mellitus (GDM), defined as impaired glucose regulation with onset or first recognition during pregnancy,^18^ is the most common obstetric complication worldwide, affecting approximately 14% of pregnancies globally.^19^ Women with GDM are at the increased risk of cesarean delivery due to a higher likelihood of adverse neonatal outcomes such as macrosomia.^20^ Despite this, current guidelines lack evidence-based recommendations for optimizing preoperative fasting protocols in this high-risk population, particularly regarding the use of carbohydrate supplementation.^21^

Therefore, this study aimed to address this gap by comprehensively evaluated the safety and the effects of preoperative carbohydrate beverage intake on maternal and neonatal metabolic responses and clinical outcomes in women with GDM undergoing cesarean delivery, compared with normoglycemic pregnant women. We assessed perioperative glycemic measures, insulin sensitivity, comfort scores, inflammatory biomarkers, postoperative recovery parameters, and maternal-neonatal outcomes, as well as metabolomic profiles.

## Results

### There was no difference in characteristics among subgroups

A total of 191 individuals were initially screened for eligibility. Of these, five participants (two with GDM and three without it) met the withdrawal criteria and were excluded. Consequently, 186 women completed the study. Clinical characteristics are summarized in Table S1. No significant differences were observed between the fasting and CHO groups within either the normal or GDM cohorts regarding age, body weight, height, body mass index (BMI), gestational age, duration of cesarean section, intraoperative fluid infusion, blood loss, or urine volume (all *p* > 0.05).

### Subjective comfort levels were increased by carbohydrate intake

Compared with the fasting groups, participants in the CHO groups from both the normal and GDM cohorts reported significantly reduced thirst, hunger, and anxiety at T2, as well as reduced thirst, hunger, nausea, and fatigue at T3 (all *p* < 0.05). A significant reduction in anxiety at T3 was also observed in the normal-CHO subgroup relative to the normal-fasting subgroup (*p* = 0.009). These findings suggest that carbohydrate intake significantly improved perioperative comfort in the pregnant women (Table 1).

**Table 1:** Maternal perioperative subjective comfort scores

		Normal			GDM		
		Fasting	CHO	P	Fasting	CHO	P
		(*n* = 45)	(*n* = 61)		(*n* = 37)	(*n* = 43)	
1 h before operation (T2)	thirst	4.31 ± 3.42	1.41 ± 2.37	<0.001	2.43 ± 2.44	1.23 ± 2.30	0.027
	hunger	4.29 ± 3.34	1.56 ± 2.50	<0.001	2.65 ± 2.45	1.19 ± 2.28	0.007
	anxiety	4.47 ± 3.29	1.56 ± 2.42	<0.001	3.08 ± 2.67	1.56 ± 2.35	0.008
	nausea	0.29 ± 1.39	0.26 ± 1.28	0.919	0.35 ± 1.53	0.00 ± 0.00	0.171
	fatigue	0.22 ± 1.22	0.23 ± 1.16	0.975	0.86 ± 2.32	0.12 ± 0.45	0.061
2 h after operation (T3)	thirst	3.13 ± 3.08	0.72 ± 1.84	<0.001	2.27 ± 2.38	0.74 ± 1.88	0.002
	hunger	3.11 ± 3.00	0.77 ± 1.88	<0.001	2.22 ± 2.35	0.70 ± 1.95	0.003
	anxiety	0.89 ± 1.76	0.15 ± 0.57	0.009	0.89 ± 1.70	0.40 ± 1.62	0.185
	nausea	1.44 ± 2.46	0.28 ± 1.13	0.004	1.38 ± 2.28	0.28 ± 1.05	0.01
	fatigue	2.02 ± 2.37	0.90 ± 2.05	0.011	1.70 ± 2.25	0.53 ± 1.01	0.005
	pain	1.44 ± 1.67	0.92 ± 1.37	0.088	1.22 ± 1.57	0.79 ± 1.32	0.191

Data are mean ± SD, *p* values are based on *t* tests.

### Blood glucose, insulin, and HOMA-IR index were not changed by carbohydrate intake

The number of samples collected at each time point was summarized in Table S2. No significant differences were found in blood glucose levels, insulin concentrations, or the HOMA-IR index between the CHO and fasting groups at T1, T2, or T4 in either the GDM or normal cohort (all *p* > 0.05). This indicates that preoperative oral carbohydrate intake did not significantly affect glycemic control or insulin sensitivity (Table S3).

### Inflammatory stress response levels were not changed by carbohydrate intake

**Figure 1:**
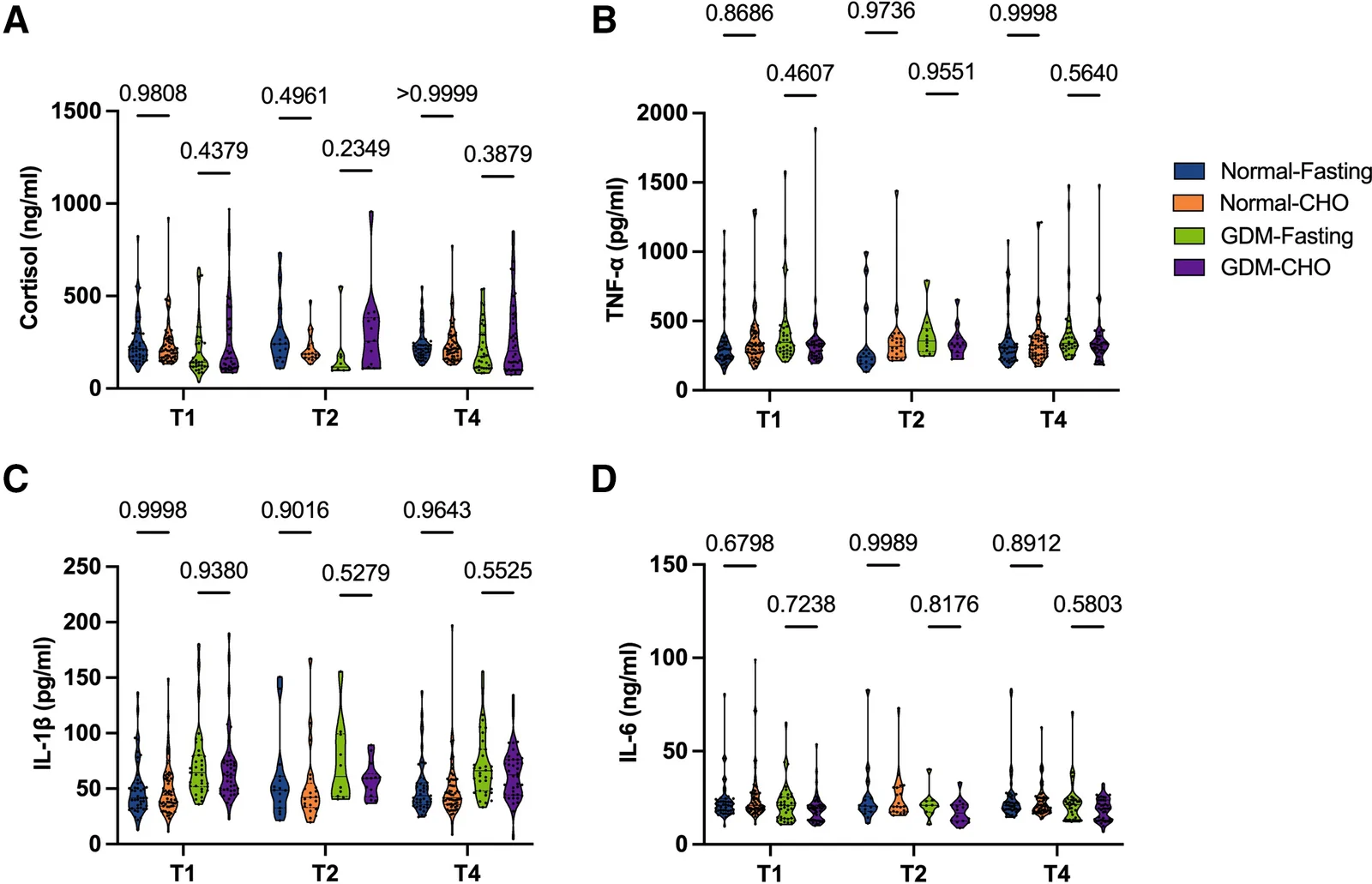
Perioperative inflammatory indicators in the fasting and CHO groups of the normoglycemic pregnant women and those with GDM (A) Cortisol, (B) TNF-α, (C) IL-6, and (D) IL-1β. T1: at admission, T2: 1 h before operation, and T4: early morning of the first day after operation. Comparisons were based on two-way ANOVA and Tukey’s multiple comparisons tests. No significant difference (*p* <0.05) was observed in the comparisons.

Plasma levels of cortisol, IL-6, IL-1β, and TNF-α were measured at T1, T2, and T4. No significant differences in these inflammatory markers were detected between the fasting and CHO groups at any time point (all *p* > 0.05, Figure 1).

### Maternal outcomes were not changed by carbohydrate intake

The incidence of intraoperative nausea, vomiting, and reflux did not differ significantly between the fasting and CHO groups in either the normal or GDM cohorts (all *p* > 0.05). No serious anesthetic complications, such as aspiration, occurred in any of the participants. Postoperatively, the two groups also showed comparable incidences of vomiting and abdominal distension (*p* > 0.05), further confirming the safety of carbohydrate administration in women with GDM undergoing cesarean delivery. Additionally, no significant differences were observed between the fasting and CHO groups in the time to first postoperative liquid diet, first flatus, first ambulation, or the length of postoperative hospitalization (all *p* > 0.05) (Table S4).

### Neonatal outcomes were not changed by carbohydrate intake

Comparisons between the fasting and CHO groups revealed no significant differences in neonatal birth weight or gender distribution (*p* > 0.05). More importantly, no significant intergroup differences were observed in the incidence of NICU admission, neonatal hypoglycemia (defined as umbilical artery blood glucose <2.60 mmol/L according to current international guidelines^22^^,^^23^^,^^24^), or indicators of metabolic acidosis (1 min Apgar score ≤7, umbilical artery pH <7.00, lactate ≥6.00 mmol/L, or base excess < −12.00 mmol/L) (all *p* > 0.05, Table S5).

### Impact of carbohydrate intake on metabolome were slightly different between normal and GDM groups

**Figure 2:**
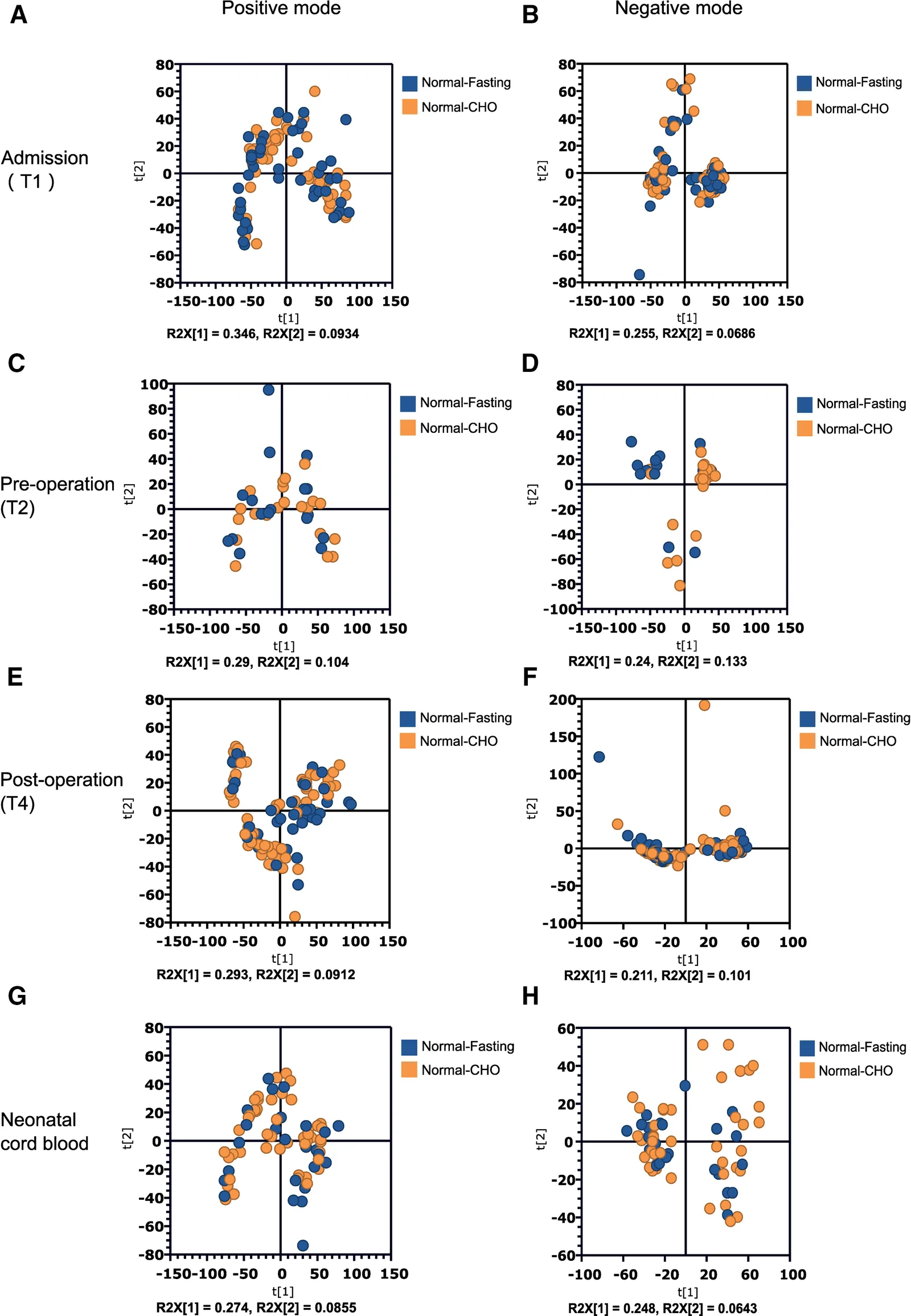
Score plots of principal component analysis of perioperative metabolomic profiles of the fasting and CHO groups of normoglycemic pregnant women (A and B) At admission; (C and D) 1 h before operation; (E and F) 1 day after operation; (G and H) neonatal cord blood. (A, C, E, and G) Positive mode; (B, D, F, and H) negative mode. Group clustering was more apparent at 1 h before operation in both positive and negative modes, indicating difference in metabolome.

**Figure 3:**
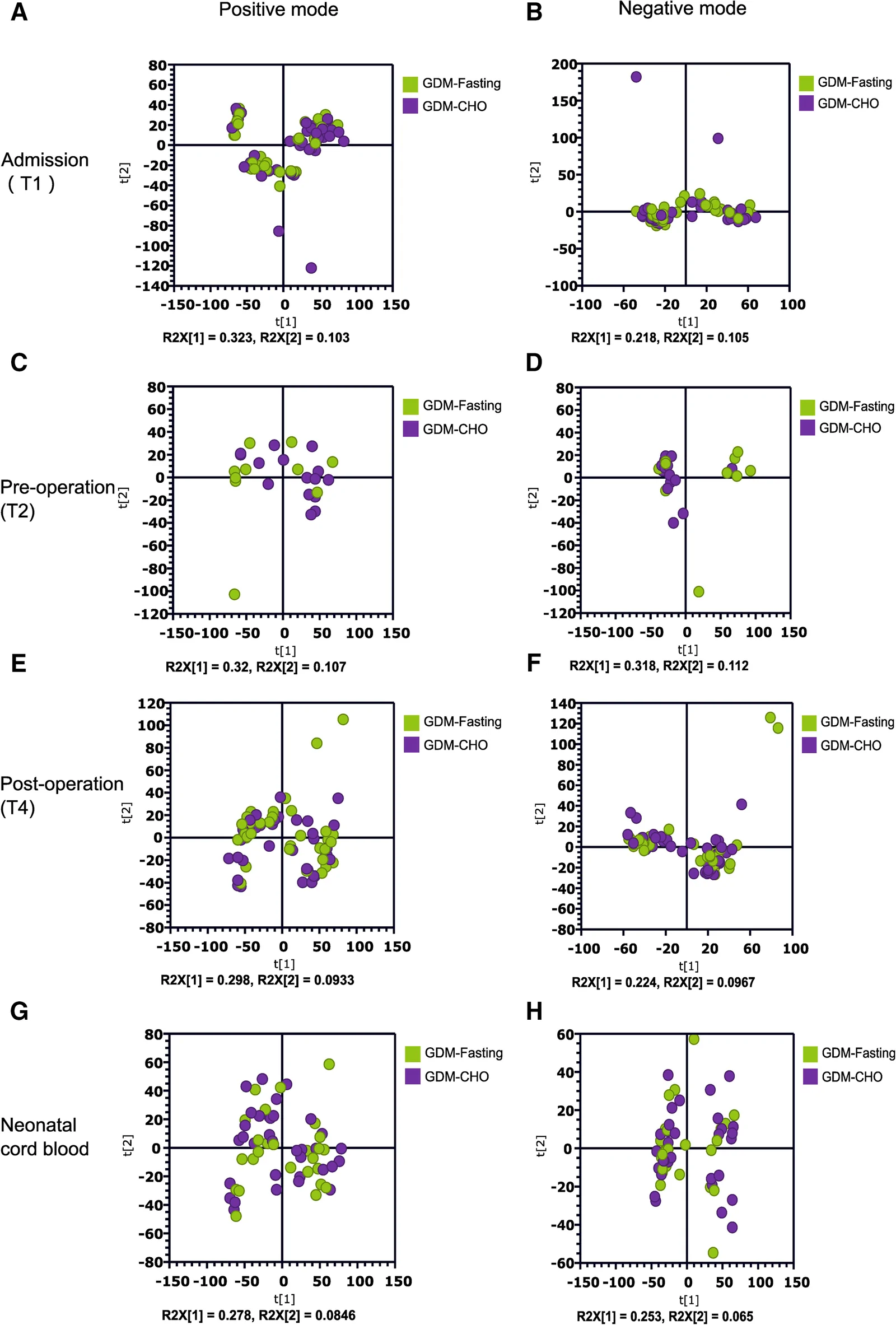
Score plots of principal component analysis of perioperative metabolomic profiles of the fasting and CHO groups of GDM patients (A and B) At admission; (C and D) 1 h before operation; (E and F) 1 day after operation; (G and H) neonatal cord blood. (A, C, E, and G) Positive mode; (B, D, F, and H) negative mode. Group clustering was more apparent at 1 h before operation in both positive and negative modes, indicating difference in metabolome.

Untargeted metabolomic analysis was performed on maternal venous blood collected at various time points and on neonatal cord blood. Principal component analysis (PCA) of maternal samples at T1 and T4, as well as cord blood samples, showed substantial overlap between the CHO and fasting groups in both ion modes. Volcano plots revealed only limited numbers of differential metabolites at these time points, indicating minimal metabolomic alterations. In contrast, pronounced metabolic differences were observed in maternal blood at T2 in both the normal and GDM cohorts (Figures 2 and 3).

**Figure 4:**
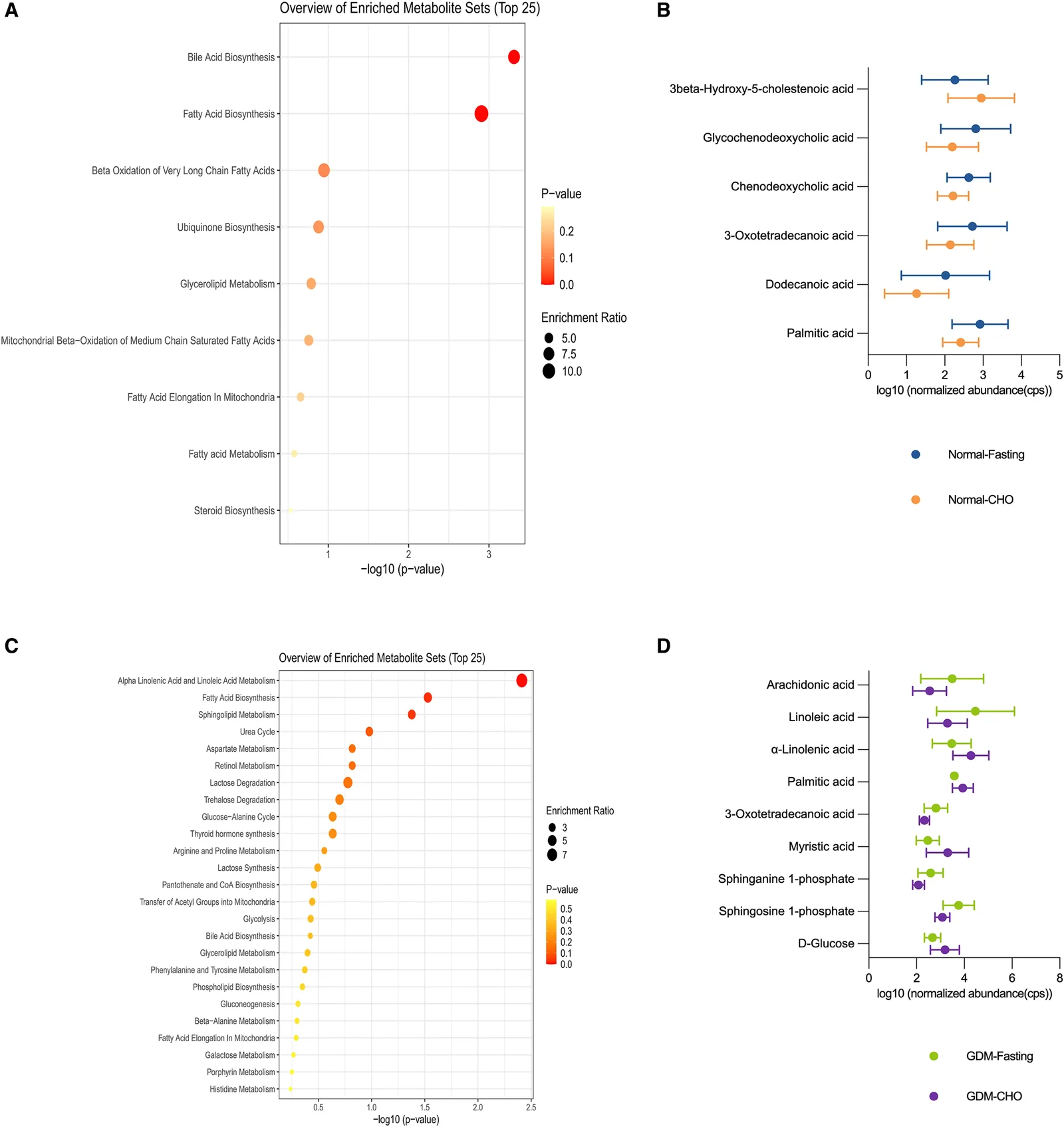
Metabolomic enrichment analysis and differential metabolites of the preoperative hour one blood of the fasting and CHO groups (A) KEGG pathway enrichment analysis of differential metabolites between fasting and CHO groups of normoglycemic pregnant women. (B) log10 of instrumental response of the significantly different metabolites (mean ± SD) between fasting and CHO groups of normoglycemic pregnant women. (C) KEGG pathway enrichment analysis of differential metabolites between fasting and CHO groups of GDM pregnant women. (D) log10 of instrumental response of the significantly different metabolites (mean ± SD) between fasting and CHO groups of GDM pregnant women.

We subsequently focused on the metabolomic profiles at T2. Given the considerable number of differential metabolites identified initially, stricter fold-change (FC) criteria were applied to pinpoint the most significantly affected metabolites. Specifically, FC ≥3 was used for comparisons in the normal group, and FC ≥4 for the GDM group. After integrating positive and negative ion data and querying the HMDB database, 14 significantly upregulated and 58 downregulated metabolites were identified in the normal-CHO group compared to the normal-fasting group. Pathway enrichment analysis revealed that six of these metabolites were involved in bile acid metabolism and fatty acid biosynthesis. In the GDM cohort, 31 metabolites were upregulated and 142 were downregulated in the GDM-CHO group versus the GDM-fasting group, with nine metabolites enriched in pathways related to linoleic acid metabolism, α-linolenic acid metabolism, fatty acid biosynthesis, and sphingolipid metabolism (Figure 4).

## Discussion

This study found that oral carbohydrate loading was not only safe but also significantly improved subjective comfort in GDM patients, modulated lipid and bile acid metabolism, and promoted preferential carbohydrate utilization. Prolonged preoperative fasting can exacerbate pre-existing insulin resistance and trigger neuroendocrine stress responses, characterized by elevated cortisol and catecholamine levels.^25^ These physiological alterations further disrupt blood glucose homeostasis, complicating perioperative glycemic management during cesarean delivery. Although oral carbohydrate loading may be both rational and safe, its use in people with diabetes—including GDM—has not been formally recommended due to concerns regarding glycemic control and associated risks.^26^ The safety data of this study are consistent with previous reports involving normoglycemic pregnant women.^16^ Preoperative carbohydrate intake did not increase the risk of intraoperative reflux, aspiration, or postoperative vomiting and abdominal distension in women with GDM. Hence, this study showed oral carbohydrate loading is also effective in GDM patients, although its impact on the preoperative maternal blood metabolome was distinct with some similarities from normoglycemic pregnant women. The carbohydrate drink used in this study was purchased by the participants at their own expense, with the dose used in this study costing approximately 360 RMB (53 USD). Although a comprehensive cost-effectiveness analysis was beyond the scope of this study, the cost may be considered relatively affordable given that the annual income in China was 43,377 RMB in 2025.^27^

The carbohydrate beverage used in this study contained high levels of sodium and glucose. Activation of co-transport of sodium and glucose via SGLT-1 in the small intestine enhances water absorption,^28^ thereby alleviating thirst related to dehydration and helping to stabilize the internal environment. Furthermore, gastric distension after fluid intake reduces ghrelin secretion and stimulates the release of gastrointestinal hormones such as GLP-1 from enteroendocrine cells. These hormones act on hypothalamic appetite centers to suppress hunger and enhance satiety.^29^^,^^30^ The rapid elevation of blood glucose and following carbohydrate intake may also help alleviate fatigue associated with hypoglycemia.^31^ Reduced sensations of hunger and thirst further modulate cortical activity, leading to suppressed HPA axis activation, lower cortisol release,^32^ and a more relaxed psychological state.

Metabolomic analyses revealed that preoperative carbohydrate intake downregulated several metabolites involved in fatty acid biosynthesis in both normoglycemic and GDM pregnancies. During fasting, the body mobilizes fat stores—releasing free fatty acids (FFAs) as an energy source and as regulators of metabolism and inflammation.^33^^,^^34^ Carbohydrate loading provides an alternative energy source, reducing the reliance on lipid mobilization and suppressing the secretion of related hormones such as cortisol and epinephrine.^35^ In addition, carbohydrates stimulate insulin secretion, which inhibits lipolysis and decreases the activity of adipose triglyceride lipase (ATGL) and hormone-sensitive lipase (HSL), thereby limiting FFA release^36^ even though a significant rise in insulin was not detected in our study. Consistent with this, inflammatory markers such as IL-6, IL-1β, and TNF-α remained unchanged, suggesting that carbohydrate supplementation may mitigate FFA-induced metabolic inflammation.^37^ Reduced fatty acid synthesis may also limit substrate availability for hepatic β-oxidation, which could theoretically decrease ketone production and reduce the risk of metabolic acidosis.^38^ This mechanism may contribute to maintaining maternal acid-base balance and protecting fetal well-being.^39^ However, ketone levels were not measured in the present study; therefore, the proposed link between altered fatty acid metabolism and ketogenesis remains speculative and should be interpreted with caution. Future studies incorporating direct measurements of ketone bodies are needed to verify this potential mechanism.

Notably, preoperative carbohydrate intake downregulated bile acid metabolism in normoglycemic women, but not in those with GDM. Bile acids are essential for lipid digestion and absorption, and their synthesis is regulated by the FGF19-FGFR4-KLB signaling axis. Postprandial FGF19 release inhibits CYP7A1—the rate-limiting enzyme in bile acid synthesis—via the FGFR4-KLB complex, thus maintaining bile acid homeostasis and preventing cholestasis or liver injury.^40^^,^^41^^,^^42^ The observed downregulation in normal pregnant women may reflect activation of this feedback circuit. In contrast, insulin resistance in GDM may impair the sensitivity of the FGF19-FGFR4-KLB pathway, blunting its regulatory effect on bile acid synthesis and resulting in a diminished metabolic response to carbohydrate loading.^43^ The different response to carbohydrate intake between normoglycemic and GDM pregnant women indicated the dose, composition and timing of carbohydrate drink may need to be optimized for GDM patients, which requires further investigation.

In conclusion, this study confirmed the safety of preoperative carbohydrate intake for both mothers and neonates, including women with normal glucose tolerance and those diagnosed with GDM. The intervention effectively alleviated perioperative discomforts such as thirst, hunger, nausea, anxiety, and fatigue, without causing significant fluctuations in blood glucose or insulin levels or amplifying inflammatory stress responses. Metabolomic analysis revealed that carbohydrate intake downregulated multiple components of fatty acid biosynthesis pathways. Additionally, in normoglycemic pregnant women, suppression of bile acid metabolism was observed 1 h before surgery. These findings supported the potential of preoperative carbohydrate loading to improve maternal comfort and promote favorable metabolic adaptations during the perioperative period.

### Limitations of the study

This study has several limitations. First, it was a single-center study, and the findings require validation through multi-center trials with larger sample sizes. Secondly, it is an observational cohort study, and participants voluntarily chose whether to receive preoperative carbohydrate loading after standardized ERAS counseling rather than being randomly assigned, which may introduce selection bias and limits causal inference. Third, postoperative blood glucose at T3, which was an important time point, was not measured. Fourth, the metabolomic analysis was exploratory, differential metabolites were identified using untargeted metabolomics without targeted validation, and the proposed mechanisms, including the potential involvement of the FGF19-FGFR4-KLB signaling pathway, remain hypothesis-generating rather than confirmatory. Future studies should include larger multicenter cohorts, evaluate individualized carbohydrate-loading strategies with different compositions, doses, and timings (with longer following-up periods), perform subgroup analyses in women with GDM, validate key metabolites using targeted approaches, and integrate complementary pathway databases to provide a more comprehensive interpretation of the underlying biological mechanisms.

## Resource availability

### Lead contact

Further information and requests for resources should be directed to and will be fulfilled by the lead contact, Chang Chen (chenchang@cqmu.edu.cn).

### Materials availability

This study did not generate new unique reagents.

### Data and code availability

• The raw data related to this paper have been deposited at Mendeley Data (https://doi.org/10.17632/8rmdznd9nr.1) and are publicly available.
• This study did not generate custom code. Analyses were performed using publicly available software packages, including SIMCA, Prism and MetaboAnalyst. Additional information regarding software and analytical parameters is provided in the STAR Methods section.
• Any additional information required to reanalyze the data reported in this paper is available from the lead contact upon request.

## Acknowledgments

The authors thank Mingjun Wu at Chongqing Medical University for assisting the LC-MS analysis. This work was supported by the 10.13039/501100001809National Natural Science Foundation of China (no.82271715 and 82071671), The 10.13039/501100013314111 Project (Yuwaizhuan [2016] 32), Chongqing Innovative Medical Device Application Demonstration Projects (Chongqing Municipal Commission of Economy and Information Technology [2024] No. 42), Chongqing Translational Medicine Center “Medicine + X” Pilot Project (Y+*X*202401), Mentor Team Project of the Discipline Peak-Climbing Program of The First Affiliated Hospital of Chongqing Medical University (CYYY-XKDFTH-DSTD-202505), and Yichang Humanwell Pharmaceutical Co., Ltd, China. The supporting sources had no involvement or restrictions regarding publication.

## Author contributions

Conceptualization, H.Z.; data curation, S.L., Y.B., X.Y., L.W., H.L., and G.S.; data analysis and visually presentation, S.L., R.H., X.T., Q.W., C.C., and H.Z.; writing – original draft, S.L.; writing-review and editing, T.W., H.Z., and C.C.; supervision, H.Z. and C.C.; funding acquisition, H.Z. and C.C.

## Declaration of interests

The authors declare no competing interests.

## Declaration of generative AI and AI-assisted technologies in the writing process

During the preparation of this work, the authors used DeepSeek in order to check grammar and polish the language. After using this tool, the authors reviewed and edited the content as needed and take full responsibility for the content of the published article.

## STAR★Methods

### Key resources table

**Table:** 

REAGENT or RESOURCE	SOURCE	IDENTIFIER
**Biological samples**		

Human plasma samples	This study	Not applicable

**Critical commercial assays**		

Human Insulin ELISA kits	Jingmei Biotechnology	JM-03944H1
Human Cortisol ELISA kits	Jingmei Biotechnology	JM-03931H1
Human IL-6 ELISA kits	Jingmei Biotechnology	JM-03204H1
Human IL-1β ELISA kits	Jingmei Biotechnology	JM-03336H1
Human TNF-α ELISA kits	Jingmei Biotechnology	JM-03277H1

**Deposited data**		

Raw data related to this paper	Mendeley Data	https://doi.org/10.17632/8rmdznd9nr.1

**Software and algorithms**		

ProteoWizard v3.0.24346	ProteoWizard	https://proteowizard.sourceforge.io
MarkerView 1.3.1	SCIEX	https://sciex.com
SIMCA v14.1.0	Sartorius	https://www.sartorius.com
Prism v10.2.3	GraphPad	https://www.graphpad.com

**Other**		

Putatively annotate differential metabolites	This paper	https://hmdb.ca
Pathway enrichment analysis	This paper	https://www.metaboanalyst.ca

### Experimental model and study participant details

This study was approved by the Ethics Committee of The First Affiliated Hospital of Chongqing Medical University (Chongqing, China; approval no. 2023-375) and registered at Medical Research Registration and Filing Information System of China (www.medicalresearch.org.cn) with the registration number MR-50-24-046877. Written informed consent was obtained from all participants prior to cesarean section. The study was conducted in accordance with the Declaration of Helsinki.

Sample size was calculated based on a previous similar study.^5^ Specifically, the effect sizes calculated from hunger and thirst scores before cesarean delivery were 0.75 and 0.57, respectively. The average effect size was then calculated to be 0.66. Using α level of 0.05 and power (1−β) of 0.8, the sample size was calculated to be 36 for each group. Considering possible drop-outs and data missing, we planned to recruit 50 participants into each group.

This observational cohort study enrolled Han Chinese pregnant women of scheduled for planned cesarean section between October 2023 and September 2024. All participants had full-term singleton pregnancies and were classified as ASA physical status class II–III.^44^ The participant flowchart was displayed in Figure S1. GDM was diagnosed based on a positive oral glucose tolerance test (OGTT) at 24–28 weeks of gestation, defined as meeting at least one of the following criteria: (1) fasting glucose ≥5.1 mmol/L; (2) 1-hour post-OGTT glucose ≥10.0 mmol/L; (3) 2-hour post-OGTT glucose ≥8.5 mmol/L. Participants with normoglycemia and without a history of metabolic disease were assigned to the Normal group. Exclusion criteria included: (1) gastrointestinal dysfunction (e.g., gastroesophageal reflux, delayed gastric emptying); (2) metabolic-related disorders such as hypertension, thyroid dysfunction, or intrahepatic cholestasis of pregnancy. Participants were also excluded if any of the following occurred: (1) cesarean section duration exceeding one hour; (2) major perioperative complications, including eclampsia, postpartum hemorrhage, amniotic fluid embolism, or postoperative transfer to the intensive care unit (ICU).

### Method details

#### Perioperative carbohydrate management

The carbohydrate drink used in this study (Yichang Humanwell Pharmaceutical Co., Ltd., China) provided 213 kJ (50.91 kcal) of energy, containing 12.5 g of carbohydrates, 50 mg sodium, 66.7 mg potassium, 63 mg phosphorus, and 65 mg chlorine per 100 mL, with an osmolality of 290 mOsmol/kg. As part of routine perioperative care, all participants received standardized counseling regarding the ERAS protocol, the decision to receive preoperative carbohydrate loading was made voluntarily by each participant after discussion with the attending clinician. Based on their OGTT results and preoperative nutritional choice, participants were classified into four groups: (1) Normal-Fasting; (2) Normal-CHO; (3) GDM-Fasting and (4) GDM-CHO. Participants who chose carbohydrate loading consumed 400 mL of the carbohydrate beverage at 22:00 in the evening before surgery, another 400 mL at 06:00 on the day of surgery, and a final 400 mL dose consumed slowly over 3 hours postoperatively. Participants who chose the fasting strategy refrained from eating and drinking from 22:00 the night before surgery until three hours postoperatively (Figure S1).

#### Indicator assessments

Blood glucose was measured at admission (T1), one hour before surgery (T2), and on the morning of postoperative day one (T4) using capillary blood and an ACCU-CHEK® Performa glucometer (Roche, Switzerland). Venous blood samples were collected at the same timepoints for analysis of plasma insulin, cortisol, IL-6, IL-1β, and TNF-α using ELISA kits (Jingmei Biotechnology, Yancheng, China), as well as metabolome.

Participants self-reported thirst, hunger, anxiety, nausea, fatigue, and pain using a visual analog scale (VAS) at T2 and two hours postoperatively (T3). Intraoperative and postoperative adverse events—including nausea, vomiting, reflux, aspiration, and abdominal distension—were recorded. Additional recovery indicators, such as time to first liquid intake, first flatus, first ambulation, and length of postoperative hospitalization, were also documented.

#### Neonatal assessment

Neonatal outcomes were assessed using medical records, including sex, birth weight, 1-minute Apgar score, and admission to the neonatal intensive care unit (NICU). Umbilical arterial blood samples were analyzed for glucose, pH, lactate, base excess (BE) and metabolomic profiles.

#### Untargeted metabolomics based on LC-MS

Plasma samples (100 μL) were deproteinized with 1000 μL of 4 °C methanol: acetonitrile (1:1, v/v), vortexed for 5 min, and incubated at 4 °C for 4 h. After centrifugation at 10,000 rpm and 4 °C for 10 min (GTR320C centrifuge, Hunan Kecheng Instrument and Equipment Co., Ltd, Changsha, China), the supernatant was evaporated to dryness using a SpeedVac SPD140DDDA vacuum concentrator (Thermo Fisher Scientific Inc., Waltham, MA, USA) at 40 °C and reconstituted in 200 μL of 50% aqueous acetonitrile. The samples were sonicated and vortexed for 5 min each, recentrifuged under the same conditions, and the final supernatant was transferred to autosampler vials.

Quality control (QC) samples were prepared by pooling equal aliquots from all plasma samples. During LC-MS analysis, one QC sample was injected after every 10 study samples to continuously monitor instrument stability and analytical reproducibility. Because of the large number of samples, analyses were performed in multiple analytical batches, and QC-based batch correction was applied during data preprocessing to minimize batch-related variation.

Chromatographic separation was performed on an LC-30A system (Shimadzu Corp., Kyoto, Japan) using an Acquity UPLC HSS T3 column (2.1 × 100 mm, 1.8 μm; Waters Corp., Milford, MA, USA) maintained at 40 °C. The mobile phase consisted of (A) 0.1% formic acid in water (positive mode) or 5 mM ammonium formate in water (negative mode), and (B) acetonitrile. The gradient program was as follows: 2% B (0 min), 20% B (1.5 min), 65% B (4.0 min), 95% B (11.0–14.0 min), 100% B (14.1–17.0 min), and 2% B (17.1–20.0 min). The flow rate was 0.4 mL/min, and the injection volume was 4 μL. Metabolome analysis was conducted on a TripleTOF 6600+ system (Sciex Corp., Marlborough, MA, USA) in independent data acquisition (IDA) mode with ESI (±). Key parameters were: IonSpray Voltage 4500 V, temperature 600 °C, GS1/GS2 60 psi, curtain gas 35 psi, and scan range m/z 80–1100.

Raw data were converted to mgf format using ProteoWizard (version 3.0.24346), and processed in MarkerView 1.3.1 for peak detection, peak alignment, retention time correction, and data normalization. Differential metabolites were selected using MetaboAnalyst 6.0^45^ with default settings and putatively annotated using the Human Metabolome Database (HMDB) 5.0^46^ with a mass error threshold below 5 ppm. Differential metabolites were screened using VIP > 1 and *p* < 0.05.

### Quantification and statistical analysis

Multivariate analysis was performed using SIMCA (version 14.1.0, Palo Alto, CA, USA), and pathway enrichment analysis was conducted in MetaboAnalyst 6.0.^45^ Univariate analyses were carried out using Prism (version 10.2.3, GraphPad Software, Boston, MA, USA). Insulin resistance was assessed via HOMA-IR, calculated as [fasting glucose (mmol/L) × fasting insulin (mU/L)] / 22.5. Normally distributed data are expressed as mean ± SD and compared using two-way ANOVA or t-test. Non-normally distributed data are presented as median (interquartile range) and analyzed with the Mann–Whitney U test. Categorical variables are expressed as n (%), and group comparisons were performed using the Chi-square test or Fisher’s exact test as appropriate. A p-value < 0.05 was considered statistically significant.

## Supplemental information

Document S1. Figure S1 and Tables S1–S5

Data S1. Registration document of the clinical trial
