# Supplementary material for: Perioperative carbohydrate loading improves comfort without adverse metabolic or neonatal effects in gestational diabetes mellitus
Source: iScience. 2026 Sep 10;29(10):117488. doi: 10.1016/j.isci.2026.117488 (PMC13586518; doi:10.1016/j.isci.2026.117488)
Supplement: Supplementary file 1 [file mmc1.pdf]

iScience, Volume *29*

Supplemental information

Perioperative carbohydrate loading improves comfort without adverse metabolic or neonatal effects in gestational diabetes mellitus

Siqi Liu (刘思琪), Yuxiang Bai (白宇翔), Xiaochang Yang (杨晓畅), Longqiong Wang (王龙琼), Hongli Li (李虹励), Guangmin Song (宋光敏), Ruohan Hu (胡若涵), Tongzhi Wu (吴同智), Xiaoqing Tan (谭晓青), Qingrong Wu (吴庆蓉), Hua Zhang (张华), and Chang Chen (陈昶)

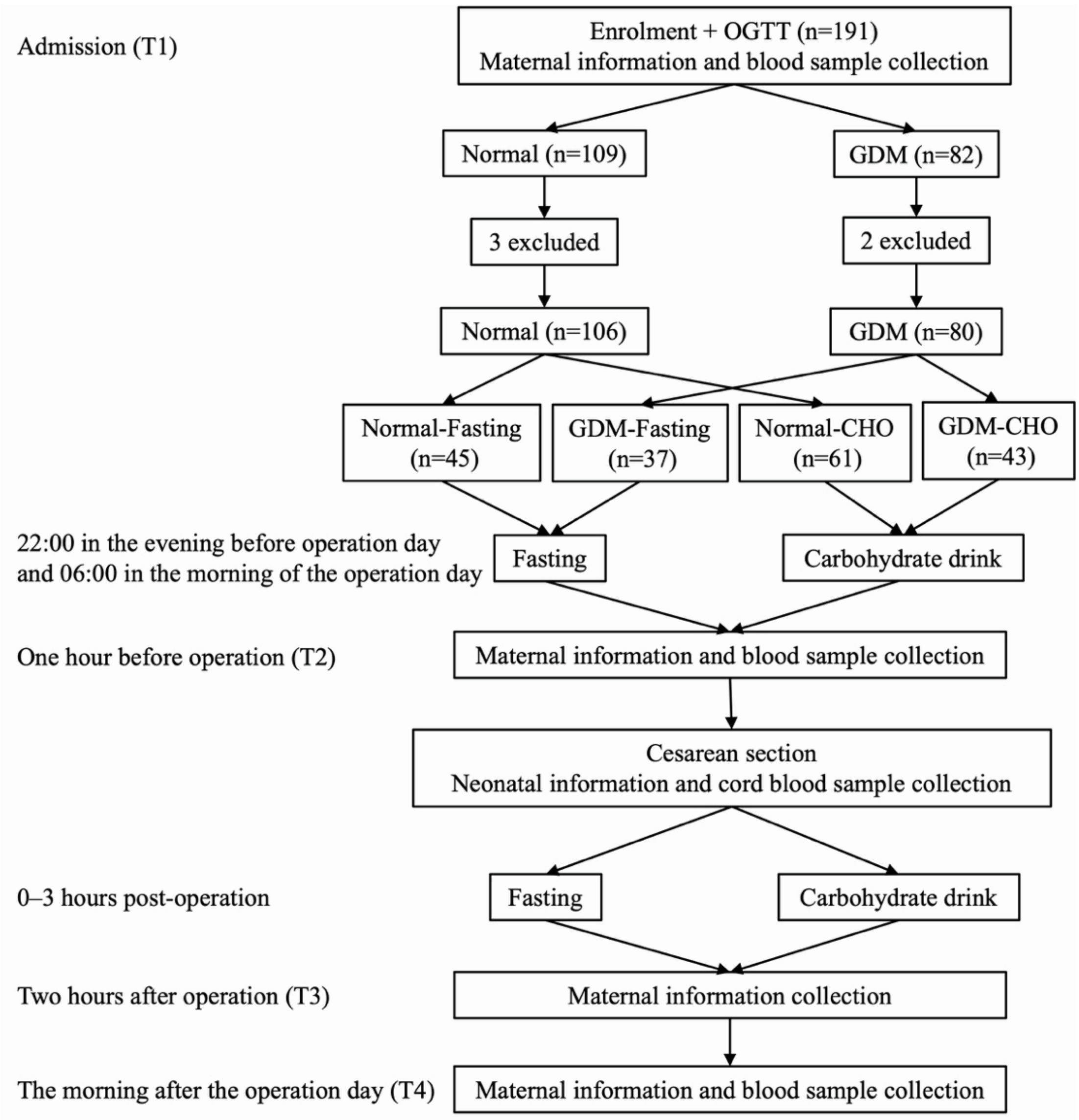

Figure S1. Participant flowchart

Table S1. Maternal demographics and clinical characteristics

	Normal			GDM		
	Fasting (n=45)	CHO (n=61)	P	Fasting (n=37)	CHO (n=43)	P
Age (years)	30.04 ± 3.93	31.46 ± 4.74	0.106	32.95 ± 4.40	31.30 ± 3.74	0.075
Body weight (kg)	53.44 ± 6.59	55.36 ± 8.47	0.21	56.26 ± 8.42	56.66 ± 9.89	0.845
Height (m)	1.60 ± 0.05	1.60 ± 0.05	0.549	1.60 ± 0.04	1.58 ± 0.05	0.085
BMI (kg/m^2)	20.95 ± 2.78	21.73 ± 3.17	0.189	21.94 ± 3.11	22.54 ± 3.33	0.412
Gestational age (week)	39.16 ± 0.88	39.13 ± 0.55	0.871	39.00 ± 0.67	38.98 ± 0.81	0.94
Duration of caesarean section (min)	40.67 ± 7.58	38.56 ± 8.60	0.193	36.19 ± 7.42	37.33 ± 7.68	0.505
Intraoperative fluid infused (ml)	1097.78 ± 209.42	1073.77 ± 220.53	0.573	1029.73 ± 256.98	1009.30 ± 220.19	0.703
Intraoperative blood loss (ml)	260.00 ± 88.93	239.34 ± 61.32	0.16	258.11 ± 74.08	227.91 ± 70.12	0.065
Intraoperative urine volume (ml)	163.56 ± 75.71	167.54 ± 64.18	0.77	139.19 ± 73.73	171.63 ± 104.01	0.117

Data are mean ± SD, P values are based on t-tests.

Table S2. Numbers of samples included in each analysis

	Normal-Fasting	Normal-CHO	GDM-Fasting	GDM-CHO
Admission (T1)	45	61	37	43
Pre-operation (T2)	17	21	10	15
Post-operation (T4)	44	61	37	41
Neonatal cord blood	25	47	26	34

Table S3. Comparisons of maternal perioperative glucose concentration, insulin concentration and HOMA-IR index

		Normal			GDM		
		Fasting (n=45)	CHO (n=61)	P	Fasting (n=37)	CHO (n=43)	P
Admission (T1)	Glucose (mmol/L)	4.85 (0.90)	4.70 (0.95)	0.301	4.90 (0.90)	5.40 (1.70)	0.087
	Insulin (mU/L)	21.68 (8.34)	22.85 (10.20)	0.305	18.28 (8.35)	17.10 (7.62)	0.404
	HOMA-IR	4.83 (2.18)	5.29 (2.43)	0.343	4.25 (2.70)	4.28 (1.93)	0.912
One hour before operation (T2)	Glucose (mmol/L)	4.60 (0.40)	4.65 (0.98)	0.503	4.90 (1.25)	5.00 (1.50)	0.361
	Insulin (mU/L)	20.49 (5.11)	21.69 (8.55)	0.297	19.88 (10.13)	17.97 (9.48)	0.579
	HOMA-IR	3.85 (3.32)	4.60 (2.96)	0.222	4.66 (2.41)	4.52 (3.45)	0.912
Early morning of postoperative day one (T4)	Glucose (mmol/L)	5.30 (1.10)	5.00 (0.85)	0.258	4.70 (0.70)	5.00 (0.85)	0.158
	Insulin (mU/L)	20.70 (8.00)	22.73 (10.04)	0.211	18.18 (9.51)	17.31 (7.99)	0.956
	HOMA-IR	5.13 (1.88)	4.94 (2.01)	0.95	3.95 (2.06)	3.67 (2.31)	0.709

Data are median (IQR), P values are based on Mann-Whitney U tests.

Table S4. Comparisons of maternal outcomes

	Normal			GDM		
	Fasting (n=45)	CHO (n=61)	P	Fasting (n=37)	CHO (n=43)	P
Intraoperative nausea (%)	13 (28.9)	18 (29.5)	0.945	9 (24.3)	9 (20.9)	0.717
Intraoperative vomiting (%)	2 (4.4)	5 (8.2)	0.709	4 (10.8)	2 (4.7)	0.537
Intraoperative reflux (%)	2 (4.4)	1 (1.6)	0.788	1 (2.7)	0 (0)	0.462
Intraoperative aspiration (%)	0 (0)	0 (0)	-	0 (0)	0 (0)	-
Postoperative vomiting (%)	9 (20.0)	7 (11.5)	0.226	8 (21.6)	5 (11.6)	0.227
Postoperative distension (%)	2 (4.4)	1 (1.6)	0.788	2 (5.4)	1 (2.3)	0.894
Time to first liquid diet (h)	19.44 ± 6.72	18.69 ± 4.89	0.698	20.27 ± 6.60	20.51 ± 3.60	0.837
Time to first flatus (h)	28.91 ± 15.93	24.62 ± 11.12	0.126	27.62 ± 12.79	25.23 ± 11.20	0.376
Time to first ambulation (h)	21.80 ± 5.34	23.75 ± 5.21	0.062	25.27 ± 8.72	23.23 ± 3.42	0.188
Length of postoperative hospitalization (h)	63.00 ± 13.36	61.74 ± 12.49	0.619	61.78 ± 13.14	62.84 ± 16.59	0.757

Data are mean ± SD with P values based on t-tests or n (%) with P values based on Chi-square tests or Fisher's exact tests.

Table S5. Comparisons of neonatal outcomes

		Normal			GDM		
		Fasting (n=45)	CHO (n=61)	*P*	Fasting (n=37)	CHO (n=43)	*P*
Infant weight (g)		3333.89 ± 333.43	3361.39 ± 334.67	0.676	3487.43 ± 399.095	3325.93 ± 445.15	0.094
Gender	Female	28 (62.2)	27 (44.3)	0.067	15 (40.5)	21 (48.8)	0.457
	Male	17 (37.8)	34 (55.7)		22 (59.5)	22 (51.2)	
NICU admission (%)		2 (3.3)	3 (6.7)	0.727	2 (4.7)	1 (2.7)	1
Hypoglycemia (%)		2（3.4）	4（9.1）	0.439	1（2.3）	2（5.7）	0.855
1 min Apgar ≤ 7 (%)		0 (0)	0 (0)	-	0 (0)	0 (0)	-
Umbilical arterial PH < 7.00 (%)		0 (0)	1 (1.7)	1	0 (0)	0 (0)	-
Umbilical arterial lactate ≥ 6.00 mmol/L (%)		0 (0)	1 (1.7)	1	0 (0)	0 (0)	-
Umbilical arterial BE < -12.00 mmol/L (%)		0 (0)	0 (0)	-	0 (0)	0 (0)	-

Data are mean ± SD with *P* values based on t-tests or n (%) with *P* values based on Chi-square tests or Fisher's exact tests.
