# Supplementary material for: Perioperative carbohydrate loading improves comfort without adverse metabolic or neonatal effects in gestational diabetes mellitus
Source: iScience. 2026 Sep 10;29(10):117488. doi: 10.1016/j.isci.2026.117488 (PMC13586518; doi:10.1016/j.isci.2026.117488)
Supplement: Supplementary file 2 [file mmc2.pdf]

DATA S1

Registration document of the clinical trial

国家全民健康保障信息平台 医学研究登记备案信息系统 ≡

张华 Zhang Hua | 项目负责人 Project Leader | 退出登录 | 帮助中心

张华 Zhang Hua
重庆医科大学附属第一医院

- 快速开始 Quick Start
- 医学研究信息上传 Medical Res
- 队列资源信息上传 Queue Reso
- 细胞治疗过程管理 Cell Thera
- 成果管理 Achievement Man
- 医学研究检索 Medical Researc
- 队列资源检索 Queue Resource
- 医学文献检索 Medical Literatu
- 临床研究课程 Clinical Researc
- 个人信息维护 Personal Inform
- 帐号管理 Account Manageme
- 登录日志 Login logs
- 使用指南 User Guide
- 免责声明 Disclaimer

项目信息 Project Information | 审核信息 Review Information

分类信息 Category Information

研究实施单位：Research Implementation Unit:	重庆医科大学附属第一医院 The First Affiliated Hospital of Chongqing Medical University
研究分类：Research Classification:	研究者发起的临床研究 - 观察性研究 Investigator-initiated clinical study - Observational study
是否是诊断试验：Is it a diagnostic trial:	否 No
暴露因素（干预措施）是否以中医理论为指导：Is the exposure factor (intervention) guided by Traditional Chinese Medicine theory?	否 No
是否为侵入式脑机接口 Is it an invasive brain-computer interface? 用于治疗神经精神疾病 For the treatment of neuropsychiatric disorders 的临床研究：Clinical research:	否 No

基本信息 Basic Information

是否需要在其他研究注册平台公开：Is it necessary in other Research Registration Platform Public:	两个平台均不公开 Neither platform is public

关闭 Close

医学研究题目：Medical Research Title:	剖宫产术前饮用多维碳水化合物对GDM孕妇及新生儿代谢的影响研究<br>A Study on the Effects of Multidimensional Carbohydrate Intake Before Cesarean Section on Metabolism in Pregnant Women with GDM and Newborns
医学研究题目简写：Brief title of the medical research:	
医学研究通俗名称：Common Name of Medical Research:	剖宫产术前饮用多维碳水化合物对GDM孕妇及新生儿代谢的影响研究<br>Study on the Effects of Multidimensional Carbohydrate Intake Before Cesarean Section on Metabolism in Pregnant Women with GDM and Newborns
本研究经费来源：Funding source for this research:	资助级别：Funding Level: 自选课题（自筹）Self-selected project (self-funded)<br>立项名称：Project Name:<br>立项编号：Project ID:<br>涉及国际合作：International cooperation involved: 否 No<br>立项时间：Project Start Date:<br>资金额度：Funding Amount: 60.0 万元 600,000 yuan
涉及材料捐献：Materials donation involved:	否 No
本研究总经费：Total research funding for this study:	60.0 万元 600,000 yuan
研究预计持续时间：Expected duration of the study:	2023-08-01 - 2025-07-01
征募研究对象时间：Recruitment period for research subjects:	-
伦理委员会：Ethics Committee:	重庆医科大学附属第一医院医学研究伦理审查委员会 Medical Research Ethics Committee of the First Affiliated Hospital of Chongqing Medical University
伦理委员会批准时间：Ethics Committee Approval Date:	2023-10-16
伦理审批件：Ethics Approval Document:	张华-2023-375-2023-10-16.pdf<br>Zhang Hua-2023-375-2023-10-16.pdf
机构立项时间：Institution project start date:	2023-10-16
机构审批通过时间：Institution approval date:	2024-11-11

实施信息 Implementation Information

数据监察委员会（DSMB）：Data and Safety Monitoring Board (DSMB):	不具有 Does not have
研究负责人：Principal Investigator:	张华 Zhang Hua
研究负责人联系方式：Principal Investigator Contact Information:	电话：023-89011798 Phone: 023-89011798
研究负责人科室：Principal Investigator's Department:	产科 Obstetrics
项目联系人：Project Contact:	

项目联系人电话：Project contact phone:

项目联系人邮箱：Project Contact Email:

项目联系人通讯地址：Project contact person's mailing address:

项目联系人所在单位：Project contact's affiliated institution:

研究团队成员：Research Team Members:

序号 No	姓名 Name	机构 Organization
1	张华 Zhang Hua	重庆医科大学附属第一医院 The First Affiliated Hospital of Chongqing Medical University

临床研究分中心：Clinical Research Subcenter: 有 There are

参与单位：Participating Institutions:

机构名称<br>Organization Name	负责人<br>Person in charge
重庆市云阳县人民医院(非系统注册的医疗机构)<br>Chongqing Yunyang County People's Hospital (Non-system registered medical institution)	向雪莲 Xue Lian Xiang
重庆市妇幼保健院 Chongqing Maternal and Child Health Hospital	文燕青 Wen Yanqing
重庆医科大学附属第二医院 The Second Affiliated Hospital of Chongqing Medical University	徐娇 Xu Jiao
重庆市人民医院 Chongqing People's Hospital	谭晓青 Tan Xiaoqing
宜宾市第一人民医院 Yibin First People's Hospital	杨凡 Yang Fan

研究状态：Research Status: 研究进行中 Research in progress

研究开始时间：Research start date: 2023-08-01

研究内容 Research Content

主要目的：Main purpose: 其他 Others

研究目的的具体描述：Specific description of research objectives:

探讨术前碳水化合物负荷对围术期患者及其新生儿代谢的影响，从而为规范使用枢能饮品、加速患者术后康复、谋取妇儿长远福利提供一定的指导依据。

To investigate the effects of preoperative carbohydrate loading on the metabolism of perioperative patients and their newborns, thereby providing a basis for the standardized use of carbohydrate-rich drinks, accelerating postoperative recovery, and ensuring the long-term welfare of women and children.

研究内容：Research Content:

于重庆医科大学附属第一医院产科行计划性剖宫产以结束妊娠的孕妇作为观察对象，根据围术期是否饮用枢能饮品分为暴露组及对照组：暴露组患者术前、术后均饮用枢能饮品，对照组患者术前、术后均未饮用枢能饮品。观察不同组别的患者在围术期不同时间段代谢水平及主观舒适度变化，记录特殊不良事件的发生；同时，观察新生儿结局及代谢水平的变化，以探讨术前碳水化合物负荷对围术期患者及其新生儿代谢的影响，从而为规范使用枢能饮品、加速患者术后康复、谋取妇儿长远福利提供一定的指导依据。

Pregnant women undergoing planned cesarean section delivery in the Department of Obstetrics at the First Affiliated Hospital of Chongqing Medical University were selected as study subjects. They were divided into an exposure group and a control group based on whether they consumed Shu-neng beverage during the perioperative period: patients in the exposure group consumed Shu-neng beverage both before and after surgery, while patients in the control group did not consume Shu-neng beverage either before or after surgery. The metabolic levels and subjective comfort changes of patients in different groups were observed at various time points during the perioperative period, and the occurrence of adverse events was recorded. Meanwhile, neonatal outcomes and changes in metabolic levels were observed to investigate the effects of preoperative carbohydrate loading on the metabolism of perioperative patients and their newborns, thereby providing a basis for guiding the standardized use of Shu-neng beverage, accelerating postoperative recovery, and promoting the long-term welfare of women and children.

学科分类：Subject Classification:

学科分类 Subject Classification
| H04 生殖系统围生医学新生儿 | H04 Reproductive System Perinatal Medicine Newborn<br>**| - H0420 妊娠及妊娠相关性疾病 | - H0420 Pregnancy and pregnancy-related diseases**

国际疾病分类(ICD-11)：International Classification of Diseases (ICD-11):

国际疾病分类(ICD-11)<br>International Classification of Diseases (ICD-11)
| 第18章 妊娠、分娩或产褥期<br>| Chapter 18 Pregnancy, Childbirth, or the Puerperium | | - L1-JA6 主要与妊娠有关的某些特指孕产妇疾患<br>| - L1-J6 Certain specific maternal diseases mainly related to pregnancy<br>| - - JA63 妊娠期糖尿病<br>| - - JA63 Gestational diabetes mellitus<br>**| - - - JA63.2 妊娠期发生的糖尿病**<br>**| - - - JA63.2 Diabetes mellitus occurring during pregnancy**
| 第18章 妊娠、分娩或产褥期 | Chapter 18 Pregnancy, Childbirth, or the Puerperium | | - L1-JA6 主要与妊娠有关的某些特指孕产妇疾患<br>| - L1-JA6 Certain specific maternal diseases mainly related to pregnancy<br>| - - JA63 妊娠期糖尿病<br>| - - JA63 Gestational diabetes mellitus<br>**| - - - JA63.Z 妊娠期糖尿病，未特指的**<br>**| - - - JA63.Z Gestational diabetes, unspecified**

具体疾病或症状：Specific disease or symptom:

妊娠期糖尿病，孕中期OGTT试验阳性（至少符合三项之二）：①空腹血糖≥5.1mmol/L；②OGTT1h血糖≥10.0mmol/L；③OGTT2h血糖≥8.5mmol/L。

Gestational diabetes mellitus, positive OGTT test in the second trimester (meeting at least two of the following criteria): ① fasting blood glucose ≥5.1 mmol/L; ② 1-hour OGTT blood glucose ≥10.0 mmol/L; ③ 2-hour OGTT blood glucose ≥8.5 mmol/L.

关键词：Keywords: 妊娠期糖尿病 Gestational diabetes; 术前碳水负荷 Preoperative carbohydrate loading; 加速康复外科 Enhanced Recovery After Surgery

研究类型：Research Type: 观察性研究 Observational study; 探索性研究 Exploratory research

是否采用额外的检查、检验、诊断措施：Are additional examinations, tests, or diagnostic measures being used? 否 No

研究阶段：Research Phase: 不适用 Not applicable

研究设计 Study Design

研究设计：Study Design: 队列研究 - 前瞻性 Cohort Study - Prospective

研究参与者性别： Research participant gender: 女性 Female

研究参与者年龄： Research Participant Age: 最小：23 岁 Minimum: 23 years old
最大：47 岁 Maximum: 47 years old

接受健康志愿者： Healthy volunteers accepted: 是 Yes

涉及弱势群体： Vulnerable groups involved: 是 Yes

弱势群体类型： Vulnerable group types: 孕期/哺乳期妇女 Pregnant/breastfeeding women

纳入标准： Inclusion criteria:

1：入选标准 （1）妊娠期糖尿病孕妇需符合以下标准：孕中期OGTT试验阳性（至少符合三项之二）：①空腹血糖≥5.1mmol/L；②OGTT1h血糖≥10.0mmol/L；③OGTT2h血糖≥8.5mmol/L。 （2）正常组孕妇需符合以下标准：孕中期OGTT试验阴性，且无任何代谢性疾病。

1: Inclusion Criteria (1) Pregnant women with gestational diabetes must meet the following criteria: Positive OGTT test in the second trimester (meeting at least two of the following three criteria): ① Fasting blood glucose ≥5.1 mmol/L; ② 1-hour OGTT blood glucose ≥10.0 mmol/L; ③ 2-hour OGTT blood glucose ≥8.5 mmol/L. (2) Pregnant women in the normal group must meet the following criteria: Negative OGTT test in the second trimester and no metabolic diseases.

排除标准： Exclusion criteria:

1：排除标准 （1）依从性差者； （2）胃肠运动功能障碍者，如胃食管返流，胃排空延迟者； （3）合并其他代谢相关疾病者，如高血压、甲状腺功能亢进、甲状腺功能减退、妊娠期肝内胆汁淤积症等。

1: Exclusion criteria (1) Poor compliance; (2) Patients with gastrointestinal motility dysfunction, such as gastroesophageal reflux, delayed gastric emptying; (3) Patients with other metabolic-related diseases, such as hypertension, hyperthyroidism, hypothyroidism, intrahepatic cholestasis of pregnancy, etc.

样本量： Sample size: 200

样本量计算依据： Sample size calculation basis: 根据本院年分娩量、GDM发病率计算，失访率按10%计算。

Based on the hospital's annual delivery volume and GDM incidence rate, the loss to follow-up rate is calculated at 10%.

是否免除知情同意： Is informed consent waived: 否 No

预期第一例研究对象签署知情同意日期： Expected date of first subject signing informed consent: 2023-09-19

研究分组信息： Research Group Information:

分组名称： Group Name: D组：未患GDM且术前未饮用"枢能"的孕妇。 Group D: Pregnant women without GDM who did not drink "Shu Neng" before surgery.

样本量： Sample size: 50

是否对照组： Control group: 是 Yes

暴露因素名称： Exposure factor name: 术前未饮用"枢能" "Shu Neng" not taken before surgery

暴露因素类型： Exposure factor type: 已批准上市的产品 - 其他产品 Approved Products - Other Products

暴露因素描述： Exposure factor description: 枢能，一种国内新兴的专为围麻醉期禁食患者定制的富含碳水化合物的特殊医学用途配方食品。饮用"枢能"：术前一天晚10：00，在半小时内饮完400ml;手术当日晨6：00，在半小时内饮完400ml；术后3小时，试饮20ml，若无不适，多次适量饮完400ml。

Shu Neng, a domestically emerging specialized medical formula food rich in carbohydrates, specifically designed for patients fasting during the peri-anesthesia period. To consume "Shu Neng": at 10:00 PM the day before surgery, finish 400ml within half an hour; at 6:00 AM on the day of surgery, finish 400ml within half an hour; 3 hours after surgery, try 20ml, and if no discomfort occurs, consume the remaining 400ml in multiple appropriate amounts.

分组名称： Group Name: B组：患GDM且术前未饮用"枢能"的孕妇； Group B: Pregnant women with GDM who did not drink "Shu Neng" before surgery;

样本量： Sample size: 50

是否对照组： Control group: 是 Yes

暴露因素名称： Exposure factor name: 术前未饮用"枢能" Did not drink "Shu Neng" before surgery

暴露因素类型： Exposure factor type: 已批准上市的产品 - 其他产品 Approved Products - Other Products

暴露因素描述： Exposure factor description: 枢能，一种国内新兴的专为围麻醉期禁食患者定制的富含碳水化合物的特殊医学用途配方食品。饮用"枢能"：术前一天晚10：00，在半小时内饮完400ml;手术当日晨6：00，在半小时内饮完400ml；术后3小时，试饮20ml，若无不适，多次适量饮完400ml。

Shuneng, a domestically emerging specialized medical formula food rich in carbohydrates, specifically designed for patients fasting during the peri-anesthesia period. To consume "Shuneng": at 10:00 PM the day before surgery, finish 400ml within half an hour; at 6:00 AM on the day of surgery, finish 400ml within half an hour; 3 hours after surgery, try 20ml, and if there is no discomfort, finish 400ml in multiple appropriate amounts.

分组名称： Group Name: A组：患GDM且术前饮用了"枢能"的孕妇； Group A: Pregnant women with GDM who consumed "Shu Neng" before surgery;

样本量： Sample size: 50

是否对照组： Control group: 否 No

暴露因素名称： Exposure factor name: 术前饮用了"枢能" Drank "Shu Neng" before surgery

暴露因素类型： Exposure factor type: 已批准上市的产品 - 其他产品 Approved Products - Other Products

暴露因素描述： Exposure factor description: 枢能，一种国内新兴的专为围麻醉期禁食患者定制的富含碳水化合物的特殊医学用途配方食品。饮用"枢能"：术前一天晚10：00，在半小时内饮完400ml;手术当日晨6：00，在半小时内饮完400ml；术后3小时，试饮20ml，若无不适，多次适量饮完400ml。

Shu Neng, a domestically emerging specialized medical formula food rich in carbohydrates, specifically designed for patients fasting during the peri-anesthesia period. To consume "Shu Neng": at 10:00 PM the day before surgery, finish 400ml within half an hour; at 6:00 AM on the day of surgery, finish 400ml within half an hour; 3 hours after surgery, try 20ml, and if no discomfort occurs, consume the remaining 400ml in multiple appropriate amounts.

分组名称： Group Name: C组：未患GDM且术前饮用了"枢能"的孕妇； Group C: Pregnant women without GDM who consumed "Shu Neng" before surgery;

样本量： Sample size: 50

是否对照组： Control group: 否 No

暴露因素名称： Exposure factor name: 术前饮用了"枢能" Drank "Shu Neng" before surgery

暴露因素类型： Exposure factor type: 已批准上市的产品 - 其他产品 Approved Products - Other Products

暴露因素描述： Exposure factor description: 枢能，一种国内新兴的专为围麻醉期禁食患者定制的富含碳水化合物的特殊医学用途配方食品。饮用"枢能"：术前一天晚10：00，在半小时内饮完400ml;手术当日晨6：00，在半小时内饮完400ml；术后3小时，试饮20ml，若无不适，多次适量饮完400ml。

Shuneng, a domestically emerging specialized medical formula food rich in carbohydrates, specifically designed for patients fasting during the peri-anesthesia period. To consume "Shuneng": at 10:00 PM the day before surgery, drink 400ml within half an hour; at 6:00 AM on the day of surgery, drink 400ml within half an hour; 3 hours after surgery, try 20ml, and if there is no discomfort, drink 400ml in multiple appropriate amounts.

是否随机分组： Randomized grouping: 是 Yes

随机分组方法： Randomization method: 简单随机 Simple random

由何人用什么方法产生随机数列： By whom and by what method was the random sequence generated: 研究者 Researcher

盲法类型： Blinding type: 开放标签，对评估者不隐藏分组 Open label, not blinded to assessors

统计分析方法描述： Statistical analysis methods description: 正态分布计量资料采用均数±标准差表示，不符合正态分布的计量资料则使用中位数和四分位间距表示。计数资料用例数 / 百分比表示。正态分布计量资料采用独立样本t检验，非正态分布计量资料采用Mann-Whitney U检验（血糖，胰岛素，胰岛素抵抗指数），计数资料采用X2检验。P＜0.05为差异有统计学意义。

Normally distributed measurement data are expressed as mean ± standard deviation, while non-normally distributed measurement data are presented as median and interquartile range. Count data are expressed as number of cases/percentage. Independent sample t-tests were used for normally distributed measurement data, Mann-Whitney U tests for non-normally distributed measurement data (blood glucose, insulin, insulin resistance index), and chi-square tests for count data. $P < 0.05$ was considered statistically significant.

测量指标： Measurement indicators:

指标名称： Indicator Name: 新生儿结局 Neonatal outcomes

类型： Type: 次要指标 - 安全指标 Secondary Endpoints - Safety Endpoints

测量时间点： Measurement time point: 新生儿娩出后 After delivery of the newborn

测量方法： Measurement method: 记录新生儿结局，如脐血糖、Apgar评分、是否转NICU；

Record neonatal outcomes such as umbilical cord blood glucose, Apgar score, and whether transferred to NICU;

描述： Description:

指标名称： Indicator Name: 代谢组学及应激产物和炎性因子浓度 Metabolomics and concentrations of stress products and inflammatory factors

类型： Type: 主要指标 - 治疗指标 - 客观化验/检查指标

Main Indicators - Treatment Indicators - Objective Laboratory/Examination Indicators

测量时间点： Measurement time point: 入院时、术前、术后第一天 At admission, preoperatively, and postoperative day 1

测量方法： Measurement method: 用质谱分析、ELISA检测以上时间段孕妇血液中的代谢物以及应激产物和炎性因子的含量。

Use mass spectrometry and ELISA to detect the levels of metabolites, stress products, and inflammatory factors in the blood of pregnant women during the aforementioned time period.

描述： Description:

指标名称： Indicator Name: 产妇结局 Maternal outcome

类型： Type: 次要指标 - 安全指标 Secondary endpoints - Safety endpoints

测量时间点： Measurement time point: 术中、术后 Intraoperative and postoperative

测量方法： Measurement method: 术中恶心、呕吐、反流、误吸，术后呕吐、腹胀、术后康复指标。

Intraoperative nausea, vomiting, reflux, and aspiration; postoperative vomiting, abdominal distension, and postoperative recovery indicators.

描述： Description:

指标名称： Indicator Name: 主观舒适度 Subjective comfort

类型： Type: 次要指标 - 治疗指标 - 主观指标

Secondary Indicators - Treatment Indicators - Subjective Indicators

测量时间点： Measurement time point: 入院时、术前、术后第一天 At admission, preoperatively, and postoperative day 1

测量方法： Measurement method: 记录孕妇术前1小时及术后2小时主观舒适度评分（即口渴感、饥饿感、焦虑感、恶心感、疲倦感、虚弱感及疼痛评分）；

Record subjective comfort scores of pregnant women 1 hour before surgery and 2 hours after surgery (i.e., scores for thirst, hunger, anxiety, nausea, fatigue, weakness, and pain);

描述： Description:

涉及生物样本采集： Involving biological sample collection: 是 Yes

生物样本： Biological samples:

样本名称： Sample Name: 血液 Blood

样本类型： Sample Type: 血液 Blood

样本去向： Sample destination: 使用后销毁 Destroy after use

说明： Instructions:

其他信息 Other Information

其他研究平台信息 Other research platform information 无 None

数据共享 Data Sharing

研究数据共享声明： Research Data Sharing Statement: 不共享 Not shared

是否公开试验完成后的统计结果： Whether to publish statistical results after trial completion: 不公开 Not public

结果发布方式： Result release method: 学术论文发表 Academic paper publication

相关附件 Relevant attachments

研究方案： Research Protocol: 研究方案.pdf Research Protocol.pdf

知情同意模板/知情同意豁免申请书： Informed Consent Template/Informed Consent Waiver Application: 知情同意书.pdf Informed Consent Form.pdf

其他文件： Other documents: 伦理审批同意书.pdf Ethics Approval Agreement.pdf

分中心 Sub-center

重庆市妇幼保健院 Chongqing Maternal and Child Health Hospital

项目负责人： Project Leader: 文燕青 Wen Yanqing

研究团队成员： Research Team Members:

序号 No	姓名 Name	机构 Organization

招募人数： Number of recruits:

招募状态： Recruitment Status:

知情同意模板/知情同意豁免申请书： Informed Consent Template/Informed Consent Waiver Application:

其他文件： Other documents:

重庆医科大学附属第二医院 The Second Affiliated Hospital of Chongqing Medical University

项目负责人： Project Leader: 徐娇 Xu Jiao

研究团队成员： Research Team Members:

序号 No	姓名 Name	机构 Organization

招募人数： Number of recruits:

招募状态： Recruitment Status:

知情同意模板/知情同意豁免申请书： Informed Consent Template/Informed Consent Waiver Application:

其他文件： Other documents:

重庆市人民医院 Chongqing People's Hospital

项目负责人： Project Leader: 谭晓青 Tan Xiaoqing

研究团队成员： Research Team Members:

序号 No	姓名 Name	机构 Organization

招募人数： Number of recruits:

招募状态： Recruitment Status:

知情同意模板/知情同意豁免申请书： Informed Consent Template/Informed Consent Waiver Application:

其他文件： Other documents:

宜宾市第一人民医院 Yibin First People's Hospital

项目负责人： Project Leader: 杨凡 Yang Fan

研究团队成员： Research Team Members:

序号 No	姓名 Name	机构 Organization

招募人数： Number of recruits:

招募状态： Recruitment Status:

知情同意模板/知情同意豁免申请书： Informed Consent Template/Informed Consent Waiver Application:

其他文件： Other documents:

© 国家卫生健康委员会科技教育司 京ICP备10218182号-22 技术支持：中国医学科学院医学信息研究所
